# Experiences of French Speaking Immigrants and Non-immigrants Accessing Health Care Services in a Large Canadian City

**DOI:** 10.3390/ijerph9103755

**Published:** 2012-10-22

**Authors:** Emmanuel Ngwakongnwi, Brenda R. Hemmelgarn, Richard Musto, Hude Quan, Kathryn M. King-Shier

**Affiliations:** 1 Health Quality Council of Alberta, 210, 811-14 Street NW, Calgary, Alberta, T2N 2A4, Canada; 2 Department of Community Health Sciences, Faculty of Medicine, University of Calgary, TRW Building, 3rd Floor, 3280 Hospital Drive NW, Calgary, Alberta, T2N 4Z6, Canada; Email: brenda.hemmelgarn@albertahealthservices.ca (B.R.H.); richard.musto@albertahealthservices.ca (R.M.); hquan@albertahealthservices.ca (H.Q.); kingk@ucalgary.ca (K.M.K.-S.)

**Keywords:** immigrants, French language, health care access, family doctor, qualitative descriptive, official language minorities

## Abstract

French speakers residing in predominantly English-speaking communities have been linked to difficulties accessing health care. This study examined health care access experiences of immigrants and non-immigrants who self-identify as Francophone or French speakers in a mainly English speaking province of Canada. We used semi-structured interviews to gather opinions of recent users of physician and hospital services (N = 26). Language barriers and difficulties finding family doctors were experienced by both French speaking immigrants and non-immigrants alike. This was exacerbated by a general preference for health services in French and less interest in using language interpreters during a medical consultation. Some participants experienced emotional distress, were discontent with care received, often delayed seeking care due to language barriers. Recent immigrants identified lack of insurance coverage for drugs, transportation difficulties and limited knowledge of the healthcare system as major detractors to achieving health. This study provided the groundwork for future research on health issues of official language minorities in Canada.

## 1. Introduction

Canada has two official languages: English and French. French is the first official language spoken in the province of Quebec, whereas English is the main language spoken in other provinces and territories, except for the province of New Brunswick which is officially bilingual. The Official Language Act [1] designates English-speakers in Quebec and French-speakers outside of Quebec as Official Language Minority Communities (OLMC). Poor proficiency in English or French negatively impacts health and access to health care services by OLMC [2,3]. The term Francophone is used to denote people whose first official language is French [4,5]. The 2006 Canadian Census revealed there were nearly 1 million Francophones residing in English speaking provinces of Canada [6]. Between 2001 and 2006, the population of Francophones in the city of Calgary, Alberta changed from 15,895 to 17,590, representing a 9.6% increase [7]. 

There are several reasons for studying experiences of minority Francophones accessing health services. First, those with limited ability to communicate in English may have difficulties accessing and using healthcare services in predominantly English speaking provinces, some of which have shortages in healthcare professionals offering services in French [8,9]. Second, the Francophone population in Calgary is very diverse, comprised of immigrants who may differ from Francophone non-immigrants in terms of culture or health care experiences prior to immigration; and they may have different experiences accessing health services. Third, minority Francophones have generally been linked to poorer health compared to residents of the same province [10,11,12]. A literature scan on this subject revealed limited research evidence to provide an understanding of factors that may contribute to a poorer health status. In an attempt to close this knowledge gap, the Canadian Institutes for Health Research (CIHR) began funding research specifically targeting OLMC [13]. 

In this light, our research team initiated a series of studies involving OLMC. The purpose of this study was to examine experiences of selected French speaking non-immigrants and immigrants accessing health services and dealing with health care in Calgary, Alberta. Understanding the issues from Francophones themselves will inform the development of targeted interventions to improve the health of this population.

## 2. Conceptual Framework

Exploring the concept of access to describe experiences with health services is important as experience may differ based on individual beliefs about health, type of service desired, point of entry, and when the service was actually utilized. Health services utilization (HSU) is often used as a surrogate indicator of health services access [14]. Researchers have used empirical explanatory or count models to conceptualize HSU among immigrants [15,16]. Unlike such mathematical models, Aday and Andersen [17] proposed a framework for the study of access to medical care. Central to this framework is the suggestion that HSU depends on three primary determinants of health behavior—the population, the health delivery system, and the external environment. Health delivery system characteristics and external environment factors are beyond individual influence, therefore not explored in this study. We suspected that population characteristics, comprised of predisposing (demographic, social structure, socio-economic position and education, and beliefs about health) and individual level enabling factors (physician supply, means and know-how), would likely mediate the experience of Francophones accessing health services. Thus, we used the Aday and Andersen framework [17] to inform our analysis and interpretation of findings, particularly focusing on enablers and detractors of healthcare access and utilization.

## 3. Methods

### 3.1. Study Design

We undertook a qualitative descriptive study to examine experiences of Francophones accessing health services and dealing with health care in Calgary, Alberta. Typically, studies using traditional qualitative methods would describe data, develop theory, or interpret phenomena [18]. We adopted a qualitative descriptive design as it is less interpretive [19]; and fit the overall aim of identifying categories to explain experiences of Francophones accessing health services.

### 3.2. Sampling

Information about our study was circulated through Francophone ethno-cultural associations; immigrant service agencies; the French Centre of the University of Calgary; and a Francophone seniors facility. We purposefully sampled participants who met specific characteristics [19,20] or certain criteria [21]. These included: (1) age 18 years or older; (2) self-identified as Francophone; (3) agreed to participate in an audio-taped interview; (4) self-identified use of family doctor (consultation with a doctor in any setting, including doctor’s offices, walk-in clinics, seniors facility) or hospital services (in-out patient, emergency) in the 12 months prior to the study; and 5) provided informed consent. 

### 3.3. Data Collection

Twenty six people (of 35 who expressed interest) participated in interviews, nine were ineligible; 23 interviews were conducted in person (at acceptable pre-arranged locations) and three interviews were conducted over the telephone. The interviews were semi-structured with open ended questions, lasted one hour or less, and were audio-recorded. Most (21) of the interviews were conducted in French.

Study participants described their experiences from the time they needed care to when they actually accessed and used health services. Sandelowski [19] refers to this data collection technique as discovering the ‘who, what, and where’ of events. We began interviews by asking participants questions to enable them to discuss the topic (*i.e.*, can you talk about your visit (to the doctor or hospital), from the moment you first thought of it, to when you made the visit, and until you left). This was followed by probing questions to help the participant focus on specific aspects of the experience, and to allow the interviewer to grasp the big picture of the experience and subsequently narrow to more detail experiences. Data collection and analysis were undertaken concurrently, which enabled us to modify interview questions and foci in order to clarify emerging themes [22].

### 3.4. Data Analysis

The interviews were interpreted and transcribed directly into English by a bilingual research assistant. Some concern has been raised about using interpreters in qualitative research. Critics point to the fact that interpreters have the potential to influence or bias how the participants’ responses are put forward [23]. To address this potential problem, the primary author (E.N.; who is also fully bilingual) reviewed the audiotapes and transcriptions of the first five interviews to confirm that initial translations were appropriate (e.g., that the translation was literally and conceptually equivalent) [24]. In addition to validating the meaning of transcripts, the primary author also participated in some of the interviews and used interview notes and tapes to provide additional clarity. 

Analysis began immediately after the first interview. Members of the research team (E.N., K.M.K.-S.) independently read interview transcripts and subsequently met to come to a consensus regarding identification of emerging themes. We used some elements of data analysis often employed in grounded theory to produce a straight descriptive summary of our data [25]. This involved line by line coding of text, otherwise known as first level coding. During this process, chunks of text that represent emerging concepts were identified and given a label. Selective or axial coding was performed next, assigning categories and condensing of first level labels. Finally each category was named and first level labels together with corresponding exemplars were summarized in tabular form based on (dis)similarity of content by immigrant status. Data collection and analysis continued until emerging themes from additional interviews revealed no new insights.

## 4. Results

As seen in Table 1, study participants (10 non-immigrants and 16 immigrants) ranged in age from 26 to 86 years. The majority of participants were married, had lived in Calgary (Canada) for less than 10 years, had obtained college or university level education, and nine out of eighteen of those who reported income, were in the upper middle [$60,000–$99,999] to highest income (>$100,000) quintiles. Amongst immigrants, 62.5% had lived in Canada for 10 years or less. 

Following analysis of transcripts, we identified four categories (access to physicians, language difficulty, interpreter services, preference for services in French) to describe individual experiences of Francophones accessing health services. 

**Table 1:** Sample distribution of participants by immigration status.

Characteristics		Total N = 26	Non immigrants (n = 10)	Immigrants (n = 16)
Age (years)				
	Mean	46.0	48.8	44.3
	Range	26–86	32–70	26–86
Sex				
	Male	14	3	11
	Female	12	7	5
Marital Status				
	Married	18	8	10
	Separated/divorced	1	1	0
	Widowed	1	0	1
	Single	3	0	3
	Common-law	3	1	2
Education				
	High school or less	5	1	4
	College or university	18	9	9
	Graduate school	3	0	3
* Income				
	<$20,000	2	0	2
	$20,000–$39,999	3	1	2
	$40,000–$59,999	4	2	2
	$60,000–$99,999	4	1	3
	>$100,000	5	4	1
	Declined	8	2	6
** Health Service Accessed in Last Yr				
	Family doctor	14	5	9
	Hospital	11	3	8
	Emergency	6	2	4
	Walk-in-clinic	4	2	2
	Other	5	0	5
Language Spoken at Home				
	French	23	9	14
	English	1	0	1
	Both English & French	2	1	1
Length of stay in Canada				
10 years or less		-	N/A	10
	Greater than 10 years	-	N/A	6

Note: Source countries for immigrants included Benin, Burundi, Cameroon, Chad, Gabon, Togo, Ivory Coast, France, Switzerland, Congo (DRC); Non-immigrants migrants came from Quebec or Manitoba. * According to the household income scale used by the Heart and Stroke Foundation of Canada [26] ** Respondents reported accessing more than one service

### 4.1. Access to Physicians

(i). *Difficulty finding family doctor*. According to our study participants, finding a family doctor, including one who speaks French was a challenging and lengthy process. One non-immigrant described it like this:

*We tried to have access to one of the doctors that we had on the list, and then it did not work out…they gave me another list and we tried again...then we went to the site on the internet [and] it was a very long process and we started with looking if we can find a doctor who speaks French…there were only family doctors who speak English on the list.*

Referring to ‘the list’ given to them by an immigrant service agency, one immigrant added:

*It is a stressful process…I went to the link [Healthlink] and they gave me a list of doctors to call, but when you call…that list is updated every six months but by the time you call, if you are lucky, and it just got updated, great...but when I got it, half of the doctors on that list couldn’t take any patients anymore…you may get lucky. *

### 4.2. Language Difficulty

Five issues related to language and communication difficulties emerged from our analysis:

(i). *Difficulty explaining self or fear of not being understood*. This issue was recurrent with both immigrants and non-immigrants. One non-immigrant explained:

*If you cannot describe what you are feeling [symptoms] it is hard for them [doctor] to diagnose anything, so I will wait. My cold will be lasting forever because I couldn’t say it was a cold or flu…I didn’t know how to describe things. I would wait until it pretty much pound my chest, and then, ok…now I really have to go.*

One participant (immigrant) illustrated difficulty with expression, by stating:

*I tried to look for a way to explain to the doctor in English for him to understand what is wrong,…‘my eyes make water’, you see, you need to have in mind to prepare in advance what you have to say to the doctor.*

Others were creative in their approach to manage this situation and used a dictionary to prepare for the conversation with the care provider.

*The day I was going to the consult, I needed vocabulary to be able to communicate; I had to look up in the dictionary to be able to know what words in French…and pronounce the words in English. *

(ii). *Emotional distress prior to visit*. For persons with language difficulties, the decision to seek care was emotional and sometimes stressful. One non-immigrant described the experience like this:

*If I am going to meet a doctor who speaks only English…I will prepare much before I go…If I can`t find a professional who can at least give me the impression of understanding my needs, I will be feeling very insecure and more timid to open up before that person. It is a relation of confidence that I want.*

(iii). *Feelings of discontent with care received.* Some participants were disgruntled with the lack of services in French, as one immigrant stated:

*I am being followed for migraine for a while now and I haven’t been able to meet somebody that speaks French and I sometimes think that it would probably have helped. I don’t know if it was a French-English thing but I am thinking if it was happening in French I probably would have been able to pass my message across.*

(iv). *Delay in seeking care*. Some participants postponed visiting a doctor until severe symptoms emerged. One non-immigrant described this experience.

*My cold will be lasting forever because I couldn’t say it was a cold or flu, I didn’t know how to describe things, I would wait until it pretty much pound my chest, and then OK now I really have to go.*

(v). *Potential for harm or medical errors.* One participant (non-immigrant) recounted a personal experience of poor communication with a provider.

*…my first delivery [of a baby], my English was very bad…at one point they gave me an I.V. [intravenous] and they said you need to press when you need some extra dose, but I didn’t understand…I don’t know if it is morphine going through to my system, I kept pushing every time, so I was druggy, I couldn’t really understand everything that was going on in the room…if my husband were not there, I would have been in trouble.*

### 4.3. Language Interpreter Services

On the whole, there was little awareness of interpretation services offered by Alberta Health Services. Yet, for those who had some experience, these descriptors and perceptions of the interpretation service emerged. 

(i). *Interpreter services are less utilized*. An immigrant described perceptions regarding lack of equal access to interpretation services.

*There is a little problem on the part of the French community who do not ask for interpretation services…and on the other hand the medical personnel do not consider the French language in an equal way as another language like Chinese for example. For a Chinese they will get an interpreter right away…but for a Francophone the medical personnel will just say oh that’s OK, we will try or I will get a nurse around who speaks French.*

(ii). *Dislike mode of translation*. An immigrant described how it is easier to speak with a translator and care provider when they are together.

*Here you have to talk to the translator she ask you to give the phone to the doctor which is, there is no three way conversation only two way conversation. And when it is like that some things are forgotten and you can’t say it all.*

(iii). *Interpreters are strangers.* There were diverse opinions about interpreter services among participants. Some thought that having an unknown third party during a visit with the doctor impacted the entire process. A non-immigrant explained the impact as follows:

*To me it is easier to talk with somebody I know and have confidence, than a translator who is more or less a stranger.*

Others thought that use of translators affect privacy which may result in them hiding some aspects of their illness. One immigrant described it like this:

*Dealing with your doctor is a very private matter which you and the doctor alone can be comfortable. With somebody else listening you wouldn’t want to deal with private issues…you end up hiding certain things then, your sickness will be hiding as well.*

(iv). *Using family and friends for interpretation*. One non-immigrant described making notes during a health care visit and ‘verifying’ information with family later. However, difficulties arose as explained:

*…with medicine, sometimes the terms are different” and this process is not ideal.*

To another participant (immigrant), taking a friend or relative to help translate and avoid making mistakes, was not ideal either.

*I bring my husband sometimes because he is Anglophone, he might know the terms but sometimes it doesn’t help. You know like we use a French term ‘migraine’ so like a little head ache, but here they use it like head problems. It is like angina.. I don’t have angina for sure. I just have a sore throat….to translate directly from French I could have said I have an angina, but it is not the same thing.*

### 4.4. Preference for health services in French

Despite reported proficiency in English, some study participants enumerated various reasons to explain their general preference for health services in French despite proficiency in English.

(i). *Feelings of entitlement and social values*. A non-immigrant described the source of these feelings like this:

*We still have hope that as a bilingual country. We can be in BC or Quebec, Alberta and still use our language…even when you are bilingual you still prefer health services in your first language, because that is the language you were brought up with.*

Another non-immigrant said “To be honest my social values enable me to live in my first language, my kids go to a French school, we try to keep our lives in French but sometimes it is impossible.”

(ii). *Same language means strong relationship with provider*. An immigrant described sharing the same language with the physician like this:

*I think it is only the language that gives you a strong relationship with the doctor…if you listen or your doctor listens to you, then you know what you are telling the doctor.*

A non-immigrant described the link between language and provider relationship like this:

*If I can’t find a professional who can at least give me the impression of understanding my needs, I will be feeling very insecure and more timid to open up before that person. It is a relation[ship] of confidence that I want.*

The non-immigrant who described the birth of her first baby used the birth of her second baby as very different—based on language compatibility.

*…with my second delivery I had a French nurse and it made a huge difference. So that was great, she could understand where I was coming from.*

(iii). *Using maternal language calms you down*. A non-immigrant put it this way:

*Health has nothing to do with the fact that you are capable of speaking the other language…when you are sick, in pain, you will want to express it in your maternal language, you will call for help in your maternal language, when you are in pain, you have a nurse who will come and talk with you in French, it will calm you down.*

### 4.5. Special Issues Related to Recent Immigrants.

Overall, we found that lack of insurance coverage for drugs, lack of transportation, and limited knowledge of the healthcare system were access issues specific to recent immigrants. Also, for some, physician gender and ability to communicate in French was the primary consideration in choosing a family doctor. 

(i). *Lack of insurance coverage for drugs*. Recent immigrants were knowledgeable about universal health insurance in Canada. However, some were concerned about not being able to meet their medication needs given that universal insurance only covers consultations. The need for additional benefits was evident.

*…universal insurance! I don’t agree…there is the Alberta Health Insurance which only cover consultations…I got a job to be able to get benefits…but the probation period was very long so I did not get the benefits by the time I left…when you are sick, they send you to the pharmacy…and you know how expensive medications are in Canada.*

This view was held by some recent immigrants some of who had been mobile, and at the time of the study were not fully employed. One participant further explained:

*I have been here for one year and I had to pay for Alberta Health Insurance…even in times when my income was not enough I had to pay.*

(ii). *Lack of transportation*. Participants for whom transportation was an issue mostly used walk-in-n clinics in lieu of family doctor. Aside from transportation, other reasons advanced for choosing walk-in-clinics included proximity of clinics to home; unstable conditions (moving from one place to another in search of a stable job); no appointments needed; and no cancellation fees in walk-in-clinics in comparison with some doctors who charge such fees for failing to meet appointments. One participant narrated.

*I always used the walk-in-clinic, the one closest to where I live. It is because I am not stable...you don’t have a well-paid job to live in the same house or apartment; sometimes you have to leave because the rent is very expensive. If you are living 20 km from your doctor and if you don’t have the means of transportation to go to the doctor, what will you do? You are obliged to use services that are closest to you.*

(iii). *Limited knowledge of the health care system*. Poor understanding of how healthcare delivery is organized impacted experiences of recent immigrants. Some intentionally delayed seeking care. One participant explained.

*…I would delay seeking care until my condition was getting worse or was lasting long because of not knowing the rules of this society.*

Another sought the wrong services—going to the emergency department (not ophthalmologist) for an eye consultation, thus placing an unnecessary burden on the emergency department. 

## 5. Discussion

We explored the experiences of Francophones in accessing health services and dealing with health care in Calgary. Overall, we found that Francophone immigrants and non-immigrants similarly experienced difficulty with finding a family doctor, English language, were reluctant to use interpretation services, and had a general preference for health services in French. But, when these barriers were overcome, their experience was much improved. For example, participants who ended up having a family doctor who was able to communicate in French reported positive experiences, were satisfied with the care received, had same doctor at the time of study and did not need interpretation services. Limited means of transportation, lack of insurance coverage for drugs and limited knowledge of the healthcare system were major detractors of access specific to recent immigrants. These findings support the applicability of Aday and Andersen framework [17] to examine experiences of Francophones accessing health services in Calgary, AB. For example, English language barrier, preference for health services in French and perceptions of interpreters, represent socio-demographic and health beliefs [in the framework] that pre-dispose Francophones to some of their health services access experiences. Likewise, difficulties finding a family doctor and limited knowledge of the health care system relate to physician supply and means and know-how, all of which are individual level enabling factors identified in the framework for health services utilization.

Most study participants reported some or good fluency in English. Language barriers or lack of services in French surprisingly, was a recurrent theme among participants; that influenced their help seeking behaviours and general perception of care received. Given the difficulty with English language, one would expect much use of language interpretation service. Instead, we observed a general preference for friends or family members over professional interpreters. Use of family or friends as interpreters can lead to medical errors with potentially serious consequences [27]. The process for interpretation, though streamlined for the health system, seemed more problematic for the participants who made comment.

The finding that study participants prefer health services in French and the accompanying reasons, is helpful to understand the ongoing discussion on health services in ‘own language’ for OLMC. Current debate on this topic is mix and often heated. There is evidence that having a health care provider who speaks the patient’s own language improves patient understanding and adherence to regimens [28]. Having services in French was perceived differently by participants. Non-immigrants seemed to feel entitled to healthcare services in French given that French is one of the official languages of Canada, and that this was a right provided for in the Constitution of Canada. Most thought it was absolutely necessary for these services to be developed, and argued that if French schools could be successfully developed and supported in an English speaking province such as Alberta, then so too should healthcare. However, a federal mandate requiring services in both official languages may not apply to healthcare delivery as healthcare in Canada is a provincial and not federal responsibility. In contrast, immigrants however, had come to Canada understanding that it is a bilingual country and expecting to communicate and integrate wherever they live without language difficulties. To them, having health services in French will be beneficial.

Transportation difficulties and lack of knowledge of the healthcare system identified by recent immigrants in this study have been documented by other researchers [29]. Not surprisingly, recent unemployed immigrants reported lack of insurance coverage for drugs. In Canada, prescription drug coverage is provided for by public or private plans. Whereas, the public plans cater for certain groups (for example seniors); private plans allow individuals to obtain coverage from employers [30]. Often, this is subject to meeting certain conditions such as completing probation or full employment. The salient access issues identified by recent immigrants highlight the realities of establishing oneself in a new country, a major challenge to the process of migration [31]. There is evidence that barriers to employment influence integration and resettlement of immigrants in Canada [32,33]; and in some cases has a negative impact on health [34,35]. Putting such issues in context helps explain why some immigrants may have certain experiences with care and not others. Our study has implications in the context of discussing health services for Canada’s French speaking minorities, who have been suggested to have poor health [36]; and less access to healthcare services than Anglophones [10]. This study demonstrated that Francophones in Calgary do access healthcare services. However, lack of services in French, compounded by a general preference for health services in French are important factors that mitigate the healthcare experience, impacts healthcare seeking decisions of Francophones, and could influence overall perception of care. A major limitation of this study is the fact that we did not consider health delivery system characteristics and external environmental factors in explaining health services access experiences of Francophone immigrants compared to non-immigrants. These, together with population characteristics constitute three primary determinants of health behavior that is central to understanding health services utilization in the Aday and Andersen framework [17]. This warrants further exploration in future research.

## 6. Conclusions

This study examined health services access experiences of Francophones in Calgary, AB. We found that immigrants and non-immigrants similarly experienced difficulty with finding a family doctor, English language, were reluctant to use interpretation services, had a general preference for health services in French. While this study has shown that health services in French would be desirable by both immigrants and non-immigrants, managing expectations for such services, providing informative resources in French as well as education regarding availability and advantages of using professional interpreters; and for recent immigrants, a better integration with settlement services, would likely be far more cost effective. Exploring both patient-provider experiences in a future study would enhance Francophone’s access and use of health services.
